# Determination of the Weight Percent of Aromatic Compounds in a Heavy Fuel Oil by Using Flash Chromatography and Solid‐phase Extraction Coupled With High‐Temperature Two‐Dimensional Gas Chromatography and Electron Ionization Time‐of‐Flight High‐Resolution Mass Spectrometry

**DOI:** 10.1002/jssc.70341

**Published:** 2025-12-28

**Authors:** Kawthar Z. Alzarieni, Wan Tang Jeff Zhang, Brent Modereger, Wanru Li, Gozdem Kilaz, Hilkka I. Kenttämaa

**Affiliations:** ^1^ Jordan University of Science & Technology Faculty of Pharmacy, Department of Medicinal Chemistry and Pharmacognosy Irbid Jordan; ^2^ James Tarpo Jr. and Margaret Tarpo Department of Chemistry Purdue University West Lafayette Indiana USA; ^3^ Department of Engineering Technology Purdue University West Lafayette Indiana USA

**Keywords:** aromatic content, fractionation, marine fuel, residual fuel oil

## Abstract

A chromatography/mass spectrometry method is presented for the determination of the weight percent of aromatic compounds in a heavy marine fuel oil. This information facilitates the estimation of the stability, engine performance, and environmental as well as health‐related consequences related to heavy fuel oils. These fuel oils are complex mixtures of many different compound types. Thus, a previously reported method was used to first separate the oil sample via distillation, precipitation, and fractionation into asphaltenes, heavy saturated hydrocarbons, alkylaromatic hydrocarbons, aromatic and heteroaromatic, as well as polar compounds. For the fractionation, both flash column chromatography and solid‐phase extraction techniques were utilized to ensure proper separation of the compound classes in the maltenes fraction of the sample. High‐temperature two‐dimensional gas chromatography coupled with high‐resolution electron ionization time‐of‐flight mass spectrometry was used to determine the overall compound compositions of the obtained fractions (other than the asphaltenes) and to classify the detected compounds. The results indicate that the saturated compound fraction contained only alkanes, while all the other fractions contained aromatic compounds. Combining the weight percent of these fractions was used to determine the weight percent of the aromatic compounds in the oil sample.

## 1 Introduction

Heavy fuel oils (HFOs) are the most widely used marine fuels [1]; virtually all medium‐ and low‐speed marine diesel engines are designed for HFOs [2]. HFOs contain elevated levels of aromatic compounds, particularly polycyclic aromatic hydrocarbons (PAHs) and alkylated aromatic compounds, as well as sulfur‐ and metal‐bearing compounds [3, 4]. During combustion, these constituents contribute to hazardous emissions, including black carbon, organic carbon, metal‐rich ash, and gaseous sulfur oxide (SO_x_) and nitrogen oxide (NO_x_), which can form secondary fine particulate matter with a diameter of 2.5 micrometers or less (PM_2.5_) and adversely affect air quality and human health, potentially leading to respiratory diseases such as asthma and increased cancer risk [5]. Regulations have been introduced to govern the permissible pollutant content in marine fuels, with the intention of reducing emissions generated during shipping, particularly from vessels propelled by HFOs [6, 7]. Until recently, regulatory attention has focused on the sulfur content of marine fuels, culminating in the 2020 International Maritime Organization (IMO) global sulfur cap, which reduced the maximum allowable sulfur concentration in HFO from 3.5% (w/w) to 0.5% (w/w) [7]. In addition, IMO Tier III standards, which entered into force in 2016, set strict limits on NO_x_ emissions from marine diesel engines operating within designated Nitrogen Emission Control Areas (NECAs), including the North American, U.S. Caribbean, Baltic Sea, and North Sea regions [8]. While sulfur content in HFO primarily affects emissions and corrosion [9], the total aromatic content can influence combustion behavior. Large marine engines increasingly use HFOs with greater carbon and aromatic fractions, which can influence ignition quality and lead to operational concerns due to incomplete combustion and coke formation [10]. Therefore, new analytical methods are needed to facilitate the determination of the aromatic compound content in HFOs.

Many spectroscopy techniques have been used to explore the aromatic compound content in fuels [11], including Fourier‐transform infrared spectroscopy (FT‐IR), proton nuclear magnetic resonance spectroscopy (NMR; for the determination of the amount of hydrogen atoms bound to aromatic rings), and fluorescent indicator absorption spectroscopy (FIA; for the determination of the total content of aromatic compounds) [12, 13, 14]. However, these techniques have limitations. For instance, FT‐IR analysis of aromatic compounds in HFOs is often compromised by complex matrix effects and baseline distortion. Additionally, spectral overlap and low specificity make reliable analysis challenging without complementary methods [13]. NMR techniques, especially standard ^1^H/^13^C measurements, fail to fully resolve the wide variety of substituted polyaromatic structures in HFOs [12]. Additionally, the high viscosity and presence of paramagnetic compounds in heavy fractions lead to line broadening and loss of signal intensity, further limiting accurate compositional breakdown for complex hydrocarbon mixtures [12, 15]. FIA spectroscopy tends to underestimate the total aromatic content in HFOs because it relies on a naphthalene‐equivalent calibration, which fails to account for the broad range of absorptivities across multi‐ring and alkyl‐substituted aromatic compounds common in such mixtures [16]. Moreover, FIA is constrained to a limited boiling point range because analytes above the gasoline range cannot be evaporated efficiently under the operational temperature required to preserve the fluorescent dye integrity [17]. Additionally, the silica‐fluorescent dye interactions lead to poor quantitation of large aromatic compounds, a limitation highlighted when comparing results obtained using this approach to more refined techniques, such as high‐performance liquid chromatography coupled with mass spectrometry (HPLC/MS) or gas chromatography coupled with mass spectrometry (GC/MS) [16, 18]. These limitations have led many organizations, including the Technical Committee of the European Committee for Standardization, to call for methods to replace FIA spectroscopy for the analysis of fuels [19].

Separation of the HFO components into fractions containing different types of compounds allows for gravimetric analysis of each fraction and consideration of mass balance‐facilitated molecular profiling of the fuel [20]. The aromatic content in HFOs is often determined by using traditional gravimetric column chromatography to separate the aromatic compounds from all other compounds, followed by analysis using a variety of analytical techniques [21]. The Saturates, Aromatics, Resins, and Asphaltenes (SARA) method has been employed for the separation of HFOs into four fractions, as the name implies [22]. However, this fractionation method often has poor reproducibility, largely because variations in solvent choice (e.g., *n*‐pentane vs. *n*‐hexane), column packing, and elution conditions lead to significant overlap between the different fractions [23]. Therefore, the fractions are complex mixtures of compounds and not separate chemical compound classes. For instance, the fraction referred to as saturated hydrocarbons contains a large amount of aromatic compounds [20, 24]. Moreover, the method is labor‐intensive and time‐consuming, which hinders its practicality for high‐throughput or routine analysis [23].

To improve the analytical reliability and prevent complications, researchers commonly prefer removing asphaltenes before analysis to avoid irreversibly adsorbing these very polar compounds into a chromatographic column [25]. The asphaltenes fraction is often precipitated from HFO by using a nonpolar hydrocarbon solvent, leaving the remaining fractions (maltenes) to be subjected to chromatography [26, 27].

GC coupled with flame ionization detection (FID) has been used for the determination of fuel contents [28]. However, the method ultimately falls short in delivering compound‐specific identification, since FID offers only bulk carbon‐sensitive signals without structural specificity [29], necessitating the adoption of MS [20].

MS has rapidly become the gold‐standard for molecular profiling of crude oil and petroleum products [30]. Although issues related to competition for ionization and matrix effects arise during mass spectrometric analysis of complex mixtures, such issues are reduced when the mass spectrometry analysis is preceded by a chromatographic separation [20]. In the last two decades, two‐dimensional GC (GC × GC) coupled to electron ionization (EI) high‐resolution time‐of‐flight MS (TOF HRMS) has been widely used for the characterization of petroleum‐based mixtures [31]. The exceptional peak capacity of GC × GC minimizes coelution of analytes; however, unresolved peaks due to co‐eluting heavy, nonvolatile compounds can still occur [32]. To address this, high‐temperature columns are often employed in GC × GC systems, extending the applicability of the method to less volatile compounds [33, 34].

The distillation (D), precipitation (P), and fractionation (F) (DPF) method was previously utilized for the determination of the gravimetric content of different types of compounds in crude oils and condensate oils of different origins [35, 36]. This method involves the initial vacuum distillation of crude oil at room temperature to remove the volatile hydrocarbons that are condensed and collected. Next, the asphaltenes are precipitated by adding *n*‐hexane under sonication. The remaining compounds, maltenes, are separated into three fractions by using automated flash chromatography: heavy hydrocarbons, aromatic and heteroaromatic compounds, and other polar compounds. The heavy hydrocarbon fraction is further separated by solid‐phase extraction (SPE) into two fractions, one containing the heavy saturated hydrocarbons and the other the light aromatic hydrocarbons [24].

In the current study, the DPF method [20, 36] was utilized for the first time for the fractionation of an HFO sample to determine the weight percent of aromatic compounds. After removing asphaltenes, the protocol uses automated flash‐column chromatography followed by SPE using pre‐packed cartridges. All elution steps occur unattended, which dramatically reduces hands‐on time and minimizes operator variability compared to traditional manual fractionation methods. Two‐dimensional high‐temperature GC × GC coupled with EI/TOF HRMS was used to confirm the successful separation of the aliphatic and aromatic compounds.

## 2 Materials and Methods

### 2.1 Materials

*n*‐Hexane (HPLC grade), dichloromethane (HPLC grade), 2‐propanol (HPLC grade), and high‐purity carbon disulfide (≥99%) were purchased from Sigma‐Aldrich, USA. Henicosane (C_21_H_44_) (98%), pentacosane (C_25_H_52_) (99%), triacontane (C_30_H_62_) (98%), and pentatriacontane (C_35_H_72_) (98%) model compounds were also purchased from Sigma‐Aldrich, USA. All chemicals were used as received without further purification. Whatman PTFE membrane filters (TE 36) were purchased from GE Healthcare Life Sciences, USA. Solid phase extraction kit (Si/CN‐S‐1.5 g) was purchased from Interchim, France. RediSep Rf Gold normal phase silica flash columns (40 g) were purchased from Teledyne Isco, USA. The HFO sample (residual fuel oil, ID 19235; CAS 68476‐33‐5) was sourced from Neste Oyj (Espoo, Finland).

## 3 Methods

### 3.1 DPF Method

The compounds in the HFO were fractionated into different chemical classes by using the DPF method [20, 24, 36]. The DPF method was performed as previously described to fractionate the HFO into six major compound classes: volatile hydrocarbons, asphaltenes, heavy saturated hydrocarbons, alkylaromatic hydrocarbons, heteroaromatic compounds, and polar compounds. The HFO was first vacuum distilled at room temperature to collect the volatile hydrocarbons, which typically have boiling points lower than 170°C. No such compounds were detected. After distillation, the remaining sample was mixed with 10 times its volume of *n*‐hexane to induce precipitation of the asphaltenes. The mixture was left undisturbed overnight to allow the asphaltenes to precipitate. The mixture was filtered through a 0.45 µm Whatman PTFE membrane filter to collect the asphaltenes. The filtrate, referred to as maltenes, was chromatographically fractionated into three compound classes based on their polarity by using a Combi‐flash RF 200 auto column system (Teledyne Isco, Inc.) and three different solvents, *n*‐hexane, dichloromethane, and 2‐propanol. The *n*‐hexane‐eluted fraction contained the heavy saturated and the small alkylaromatic hydrocarbons, while the dichloromethane and the 2‐propanol fractions contained the heteroaromatic and the polar compounds, respectively. Finally, the *n*‐hexane‐eluted fraction was subjected to gravitational solid‐phase extraction by using a Si/CN‐S‐1.5 g solid‐phase cartridge (Interchim) to separate the heavy saturated hydrocarbons from the alkylaromatic hydrocarbons. The mass of each recovered compound class was determined and used in calculating the gravimetric wt% of the class relative to the mass of the sample subjected to fractionation. Gravimetric measurements were performed using a mettler Toledo XS105 semi‐micro balance with a readability of 0.01 mg for masses upto 82 g. For the 20 g samples used in distillation, this corresponds to to a practical limit of detection of approximately 0.00005 wt%, with mass changes below this threshold considered unmeasurable. Sample loss that occurred during transfer and processing was determined after each step and was excluded from the original mass of the analyzed sample. The loss ranged from 3.0% to 4.7%, which is lower than those reported in the literature for the analysis of crude oil and condensates by using the DPF method (8%−17%) [20, 35, 36], likely because the HFO samples contained very low amounts of volatile compounds, resulting in higher stability and reduced mass loss during fractionation and subsequent analysis. More details on the fractionation methods can be found in Table S1.

### 3.2 Two‐dimensional GC/EI TOF HRMS

The compound fractions, except for the asphaltenes, were analyzed using a LECO Pegasus 4D GC × GC/EI TOF HRMS instrument [37]. Each fraction was dissolved at a concentration of 1.0 wt% in a solvent appropriate to its type: CS_2_ for maltenes, *n*‐hexane for the *n*‐hexane‐eluted fraction, CH_2_Cl_2_ for the CH_2_Cl_2_‐eluted fraction, and 2‐propanol for the 2‐propanol‐eluted fraction.

An auto injector (Agilent G4513A) was used to inject 0.5 µL of the solutions into a split/splitless injector with a split ratio of 50:1 that was held at 260°C under a constant flow of helium carrier gas (1.25 mL min^−1^). The compounds were separated using a polar 60 m capillary column (ZB‐35HT Inferno; Zebron, 35% phenyl/65% dimethylpolysiloxane stationary phase; 0.25 mm inner diameter, 0.25 µm film thickness) followed by a nonpolar 2 m capillary column (ZB‐1HT Inferno; Zebron, 100% dimethylpolysiloxane stationary phase; 0.25 mm inner diameter, 0.25 µm film thickness). These columns can withstand temperatures up to 430°C. The primary oven containing the polar column was set at 100°C and was held at that temperature for 1 min. After that, the oven temperature was ramped to 330°C at a rate of 5°C min^−1^ where it was held for 5 min. A quad‐jet dual‐stage thermal modulator placed between the two columns consisted of two hot jets and two cold jets, one hot and one cold jet pair for each stage. Liquid nitrogen was used for cooling the cold jets, and heated nitrogen gas was used for the hot jets. The modulation period was set to 5.0 s throughout the entire analysis. Three different hot pulse duration times were used: 0.8 s (start of experiment to 8000 s), 1.25 s (8000 s–12,005 s), and 1.70 s (12,005 to the end of experiment). Hot pulse durations were varied in three time segments to better accommodate analytes spanning a wide volatility range, with shorter pulses applied during the elution of lighter compounds and longer pulses later in the experiment to improve desorption of heavier compounds. Such adjustment of modulation parameters is consistent with established GC × GC optimization strategies for complex mixtures [38]. The modulator temperature was 15°C greater than the temperature of the secondary oven containing the nonpolar column. The secondary oven temperature was set at a temperature 5°C greater than that of the primary oven and was subjected to the same ramp rates as the primary oven. The transfer line was held at 350°C. The MS ion source was heated to 250°C. The eluting compounds were ionized by EI using 70 eV electron kinetic energy. All ions were transferred into the TOF MS for high‐resolution analysis (25,000 resolution at *m/z* 219). Mass spectra were collected at an acquisition rate of 200 Hz. Mass calibration and tuning were conducted daily by using perfluorotributylamine as the calibration agent. To avoid saturating the detector and harming the EI filament, an acquisition delay of 300 s was employed to prevent the ionization of the solvent. Data collection, processing, and analysis were performed via LECO Visual Basic Scripting software, ChromaTOF version 5.10.58.0.52262. Wiley (2011) and NIST (2011) mass spectral databases were used for compound classification. For peak picking, a signal‐to‐noise ratio threshold of ≥ 25 and a library match factor threshold of > 800 were used [35, 36].

A prior study has highlighted the challenges in accurately identifying heavy saturated hydrocarbons by using EI MS, especially in distinguishing larger isomeric alkanes due to their extensive fragmentation and the absence of molecular radical cations [39]. However, in the present study, no classification of saturated hydrocarbons was needed, only their clean separation from the aromatic compounds. In order to facilitate this task, the EI TOF HRMS data were considered with reference to the GC retention times of standard *n*‐alkanes, providing a more reliable method to differentiate heavy saturated hydrocarbons from aromatic compounds.

## 4 Results and Discussion

The DPF method [24] was used for the fractionation of a marine HFO sample into six compound classes: volatile hydrocarbons, asphaltenes, heavy saturated hydrocarbons, alkylaromatic hydrocarbons, aromatic and heteroaromatic compounds, and polar compounds (Table 1). As expected, the HFO did not contain measurable amounts of volatile hydrocarbons [37]. Therefore, only five compound classes were retained. The original HFO sample was black in color but the colors of the fractions were found to vary, indicating that the fractions contained different classes of compounds responsible for the color differences (Figure S1). The heavy saturated hydrocarbon fraction was viscous and had a light‐yellow color. On the other hand, the fraction containing alkylaromatic compounds was transparent or white, the fraction containing aromatic and heteroaromatic compounds had a reddish‐brown color, the fraction containing the polar compounds had a white color, while the asphaltenes fraction corresponded to black solids. The average gravimetric wt. % of compounds in each compound class was determined from the total mass of each fraction relative to the mass of the sample subjected to fractionation. This analysis revealed that the most dominant fraction was the one containing the aromatic and heteroaromatic compounds, comprising 54.2% of the sample. This was followed by heavy saturated hydrocarbons at 36.1%. Asphaltenes accounted for 3.3%, while alkylaromatic compounds and polar compounds were present in smaller quantities, at 1.8% and 0.9%, respectively. Results obtained from replicate measurements and the relevant standard deviations are provided in Table 1. The relatively high standard deviations observed for the heavy saturated and the heteroaromatic fractions can be explained by the intrinsic non‐homogeneity and high viscosity of these fractions. These properties complicate not only elution and transfer during processing but also obtaining samples with identical compositions from the bulk sample. Such compositional variability is a well‐recognized feature of petroleum products, including residual fuels [40].

**TABLE 1:** Average gravimetric wt% and standard deviations (SDs) measured for the different compound classes obtained by using the Distillation, precipitation, fractionation (DPF) method on a heavy fuel oil (HFO) sample.

Chemical class	Gravimetric weight percentages			Data analysis	
	Trial #1	Trial #2	Trial #3	Average	SDs
Heavy saturated hydrocarbons	30.2 %	42.0 %	36.0 %	36.1 %	5.9
Alkylaromatic compounds	1.0 %	1.9 %	2.4 %	1.8 %	0.7
Volatile hydrocarbons	0.0 %	0.0 %	0.0 %	0.0 %	0.0
Polar compounds	0.8 %	0.9 %	0.9 %	0.9 %	0.1
Aromatic and heteroaromatic compounds	62.7 %	46.8 %	53.0%	54.2 %	8.0
Asphaltenes	2.3 %	3.7 %	3.8 %	3.3 %	0.8
Loss	3.0 %	4.7 %	3.9 %	3.9 %	0.9
Total	100.0 %	100.0 %	100.0 %	100.0 %	0.0

The fractions, except for the asphaltenes, were analyzed by using GC × GC/(EI) TOF HRMS to monitor the efficiency of the fractionation and to determine the compound types in each fraction. The analysis results for the compound classes, the average gravimetric wt. % of compounds in each compound class and the wt. % of aromatic compounds in the HFO are discussed below in this order.

**FIGURE 1:**
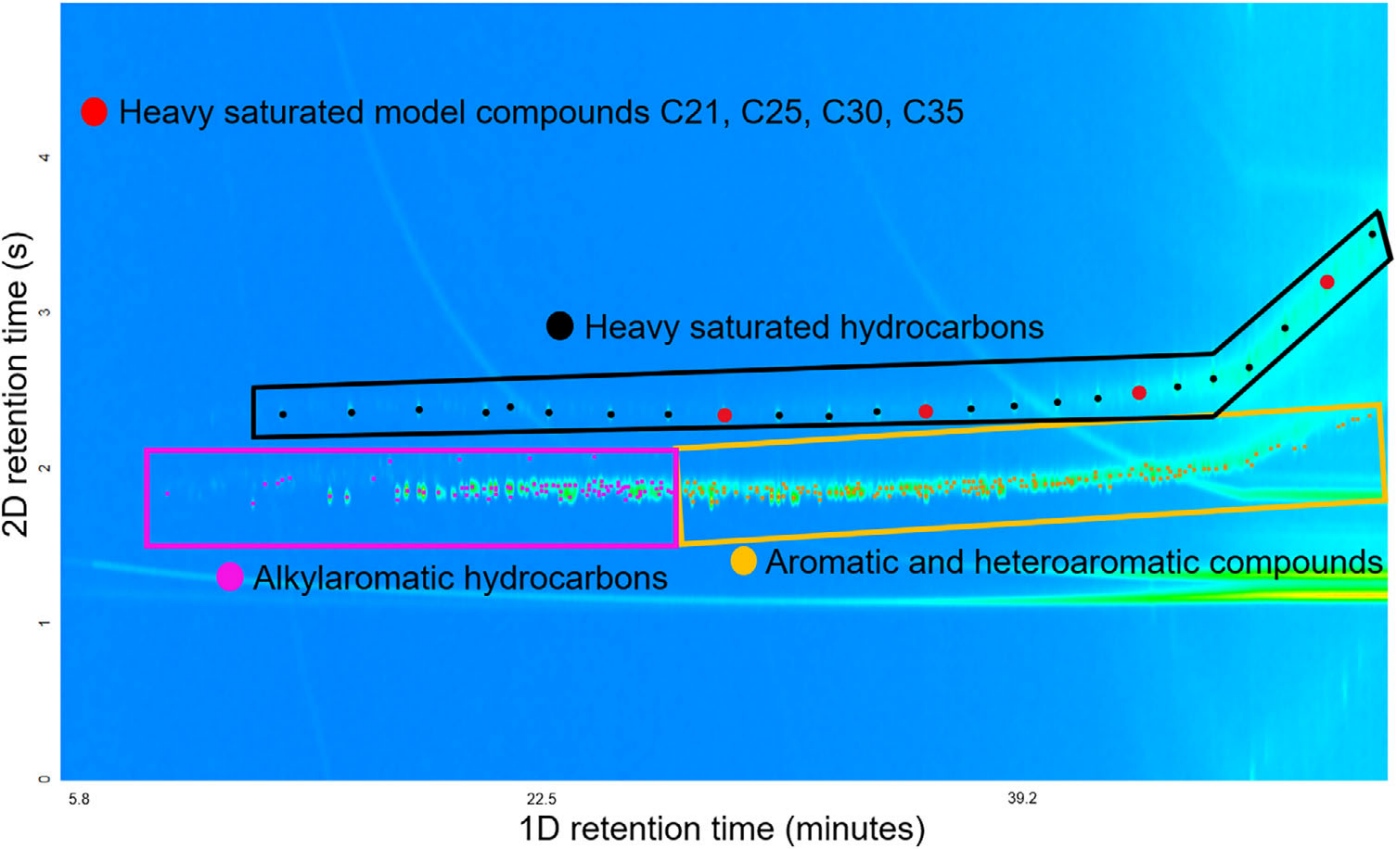
Two‐dimensional gas chromatography coupled to electron ionization high‐resolution time‐of‐flight mass spectrometry (GC × GC/EI TOF HRMS) total ion chromatogram of the maltenes derived from the heavy marine fuel oil sample. *Note*: the green streaks arise from column bleeds. This phenomenon is, unfortunately, common in high‐temperature analyses conducted via GC [44, 45].

Figure 1 shows the GC × GC/EI TOF HRMS total ion chromatogram for the maltenes fraction of the HFO. The chromatogram demonstrates that the majority of the compounds in the maltenes fall within three compound classes: heavy saturated hydrocarbons, alkylaromatic hydrocarbons, and aromatic and heteroaromatic compounds [31, 41, 42]. The indicated GC × GC elution regions have been previously delineated for these different types of compounds by using many model compounds and the same methods on the same instrumentation [42, 43]. To validate these assignments in the present work, long‐chain saturated hydrocarbon standards were injected, separately from the maltenes fraction, for analysis using the exact same GC × GC/EI TOF HRMS method to confirm that large saturated compounds elute precisely within the predicted region, thus reinforcing the previous model‐compound‐based elution region definitions.

**FIGURE 2:**
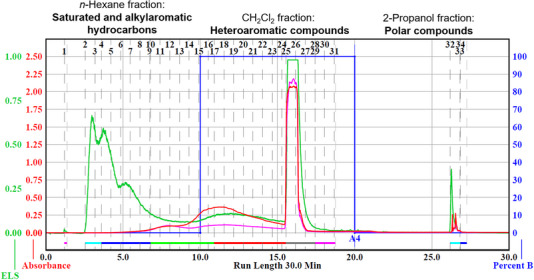
Auto column‐ELS/UV chromatogram of maltenes, indicating the separation of maltenes into three fractions. The presence of both evaporative light scattering (ELS) (green signal) and ultraviolet (UV) (red signals) signals after 5 min of eluting n‐hexane indicates the co‐elution of some aromatic compounds (UV absorption: red and purple traces) along with the aliphatic hydrocarbons (no UV absorption).

**FIGURE 3:**
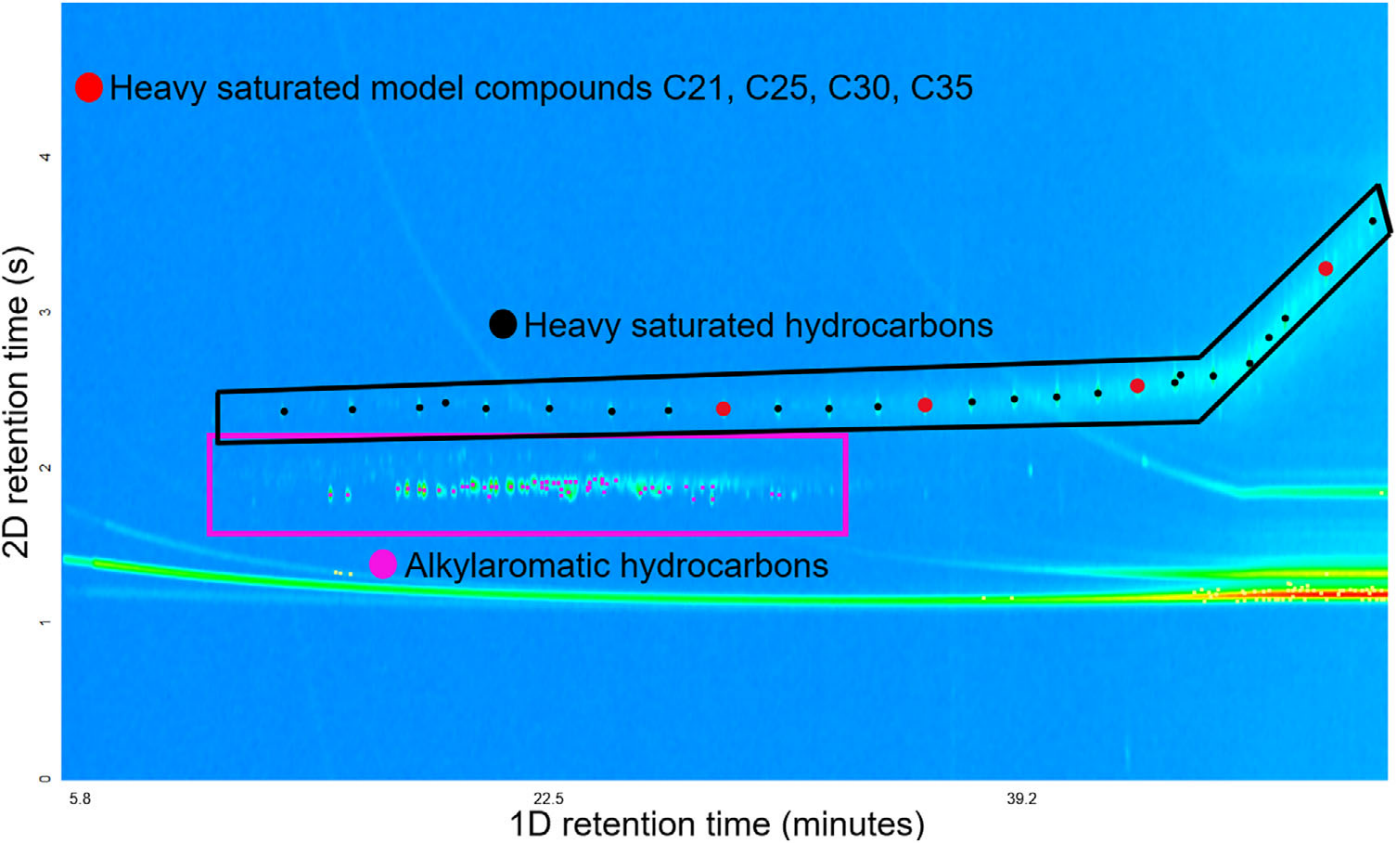
The total ion chromatogram obtained using two‐dimensional gas chromatography coupled to electron ionization high‐resolution time‐of‐flight mass spectrometry (GC × GC/EI TOF HRMS) for the compounds eluting with n‐hexane solvent from the flash column shows that the heavy saturated hydrocarbons (within black frame) and some aromatic compounds (within purple frame) had co‐eluted from the flash column. Four heavy model compounds (red circles) had been added into the analyzed mixture.

The maltenes fraction was chromatographically separated into three fractions based on the differences in the polarity of the compounds by using a flash chromatography system coupled with an evaporative light scattering (ELS) detector and an ultraviolet (UV) photodetector. The eluents used were *n*‐hexane, dichloromethane, and 2‐propanol, in this order, and each was eluted for 10 min. Figure 2 shows the chromatogram of the separated maltenes. The presence of both ELS and UV signals after 5 min of elution by using *n*‐hexane indicates the co‐elution of two different compound types, aliphatic hydrocarbons (no UV absorption) and aromatic hydrocarbons (with UV absorption). This was confirmed by GC × GC/EI TOF HRMS analysis (Figure 3). Therefore, an additional separation step was necessary.

The saturated and alkylaromatic hydrocarbon classes in the *n*‐hexane‐eluted fraction were separated further via SPE to produce fractions containing only the heavy saturated hydrocarbons and only the alkylaromatic hydrocarbons, thus increasing the total number of maltene fractions from three to four.

**FIGURE 4:**
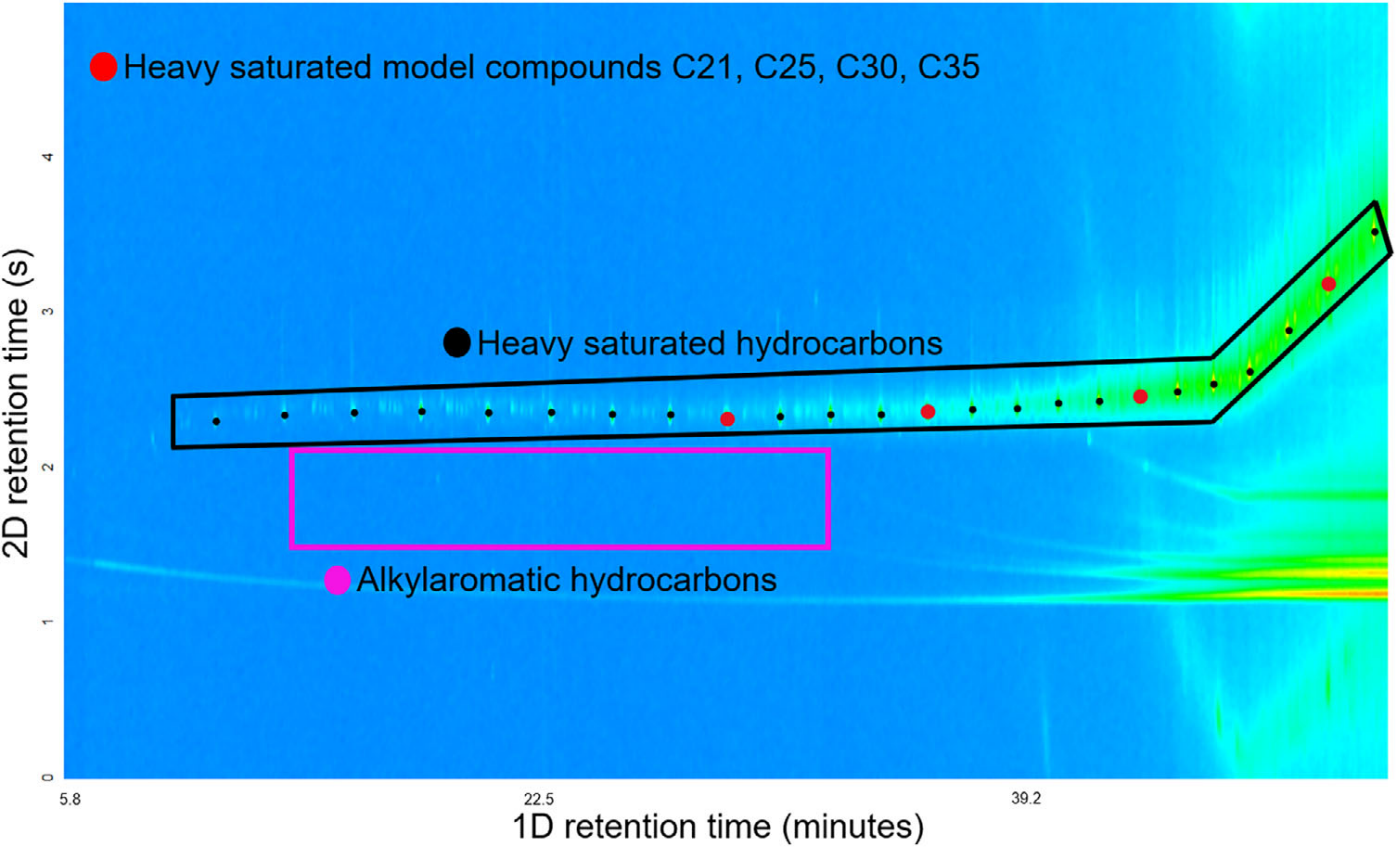
The total ion chromatogram obtained using two‐dimensional gas chromatography coupled to electron ionization high‐resolution time‐of‐flight mass spectrometry (GC × GC/EI TOF HRMS) for the *n*‐hexane SPE fraction containing only heavy saturated hydrocarbons (in black frame) without carryover from aromatic compounds (purple frame). A mixture of saturated hydrocarbon model compounds was analyzed under identical conditions. The resulting elution contours were overlaid on the maltene‐derived sample signal, with red dots marking the exact elution coordinates for each *n*‐alkane model compound.

The success of the solid phase extraction was evaluated by using GC × GC/EI TOF MS. Indeed, the total ion chromatogram measured for the saturated hydrocarbon fraction (Figure 4) indicates no presence of aromatic compounds. Based on the location of the GC × GC peaks and the best compound hits with MS library data, the majority of the compounds in the heavy saturated hydrocarbon fraction were linear saturated hydrocarbons, with a subset exhibiting minor branching.

To determine the number of carbons in the heavy saturated hydrocarbons, a 1% wt/wt solution of henicosane (C21), pentacosane (C25), triacontane (C30) and pentatriacontane (C35) model compounds in *n*‐hexane was analyzed by using the same GC × GC/EI TOF HRMS method, and the signals of the model compounds were overlayed on the signals of the compounds derived from the maltenes (Figure 4). The results indicate that the heavy saturated hydrocarbons predominantly consist of compounds with 30–36 carbon atoms, although a portion falls within the C12–C29 range, and a minor fraction contains as few as 10 carbon atoms (Table S2 in the Supporting Information).

In a previous study on the characterization of an HFO, SARA fractionation followed by GC analysis suggested that the saturated hydrocarbons contained up to 29 carbon atoms [37]. While saturated hydrocarbons with longer carbon chains might have been present in the sample, their confirmation was impeded by the use of conventional GC analyses. Furthermore, the SARA method has been noted to cause precipitation of some saturated hydrocarbons with the asphaltenes, which were found to contain approximately 2% saturated hydrocarbons as determined by elemental analysis [37]. In the present study, the saturated hydrocarbons fraction (36.1%) mostly contained compounds with 30–36 carbon atoms, which aligns with the expected dominance of large compounds in such fuels [37]. During method development, a GC × GC scout run was performed on the heavy saturated hydrocarbon fraction (Figure S2) to confirm elution of the largest analytes; based on these results, the final method parameters were chosen to ensure complete elution of all components, with no compounds exceeding 36 carbon atoms in the fraction.

**FIGURE 5:**
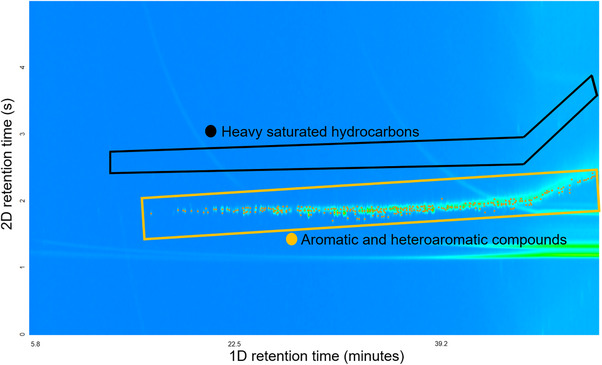
The total ion chromatogram obtained using two‐dimensional gas chromatography coupled to electron ionization high‐resolution time‐of‐flight mass spectrometry (GC × GC/EI TOF HRMS) shows that the CH_2_Cl_2_ fraction contains only aromatic and heteroaromatic compounds (yellow frame) without carryover from heavy saturated hydrocarbons (black frame).

As expected, intense UV absorbance by some compounds was observed for the fraction eluted from the flash column by using the CH_2_Cl_2_ eluent (Figure 2). This observation often indicates the presence of aromatic compounds. In order to test this hypothesis, the CH_2_Cl_2_‐eluted fraction was analyzed via GC × GC/EI TOF HRMS. The analysis revealed the presence of aromatic and heteroaromatic compounds with no evidence of carryover from heavy saturated hydrocarbons (Figure 5).

**FIGURE 6:**
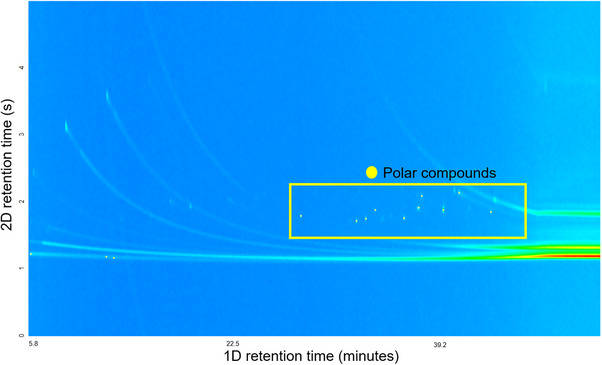
The total ion chromatogram obtained using two‐dimensional gas chromatography coupled to electron ionization high‐resolution time‐of‐flight mass spectrometry (GC × GC/EI TOF HRMS) for the flash column fraction obtained using 2‐propanol eluent contained only aromatic polar compounds (yellow frame) based on their elution region and EI‐based identification, without carryover from compounds in the other fractions.

Only a few minor compounds were noted to show ELS and UV absorption signals in the flash chromatograph when 2‐propanol eluent was employed (Figure 6). The elution behavior in GC × GC of the compounds in the 2‐propanol fraction supported their classification as aromatic. These compounds eluted rapidly from the nonpolar column and exhibited prolonged retention in the polar column, indicative of their polar, likely aromatic character. The aromatic nature of these compounds was confirmed by GC × GC / EI TOF HRMS analysis (Table S3 in the Supporting Information). Thus, they were also considered part of the aromatic compound content in the fuel. However, this fraction represented only 0.9% of the total fuel mass.

The effective separation of fuel components enabled the accurate determination of the total aromatic compound content. The saturated hydrocarbon fraction was confirmed to be free of aromatic compounds and was therefore excluded from the quantification. The remaining fractions, comprising aromatic and heteroaromatic compounds, alkylaromatic hydrocarbons, and polar compounds, were collectively considered as the aromatic compound portion of the fuel. Also, asphaltenes were assumed to be aromatic, consistent with their well‐established structural characteristics [46]. Consequently, for this specific fuel sample, the aromatic content was defined as the sum of all fractions excluding the saturated hydrocarbons at 60.2 ± 0.1%.

An ongoing study, which will be published separately on different types of samples, is focused on establishing a quantitative relationship between GC × GC/EI TOF HRMS and GC × GC/FID signals and the gravimetric content of the respective compound classes. This study will address cases wherein a mixture of different types of compounds is present.

## 5 Conclusions

The DPF method [20, 36] was successfully applied to fractionate a marine HFO sample into five distinct compound classes: asphaltenes, heavy saturated hydrocarbons, alkylaromatic hydrocarbons, aromatic and heteroaromatic compounds, and polar compounds. The use of high‐temperature GC × GC/EI TOF HRMS analysis confirmed the effective separation and classification of these different compounds. Heavy saturated hydrocarbons constituted 36.1% of the sample, establishing them as the second most dominant fraction.

Chromatographic separation and subsequent gravimetric analysis of the dichloromethane fraction indicated that aromatic and heteroaromatic compounds were the most abundant compounds in the original sample, accounting for 54.2% of the sample, alongside alkylaromatic hydrocarbons (1.8%) and asphaltenes (3.3%). The 2‐propanol fraction contained a small proportion (0.9%) of polar compounds, which were also determined to be aromatic. The total gravimetric percentage of aromatic compounds was calculated to be 60.2 ± 0.1% providing an overall assessment of the aromatic content in the HFO sample.

In summary, the DPF method has been demonstrated to facilitate the fractionation of heavy fuel oil into chemically distinct classes. This robust separation enables targeted downstream analysis of each fraction, whether by spectroscopic or spectrometric techniques. As a result, the DPF method has now been successfully validated across a broad range of feedstocks, from crude oil and condensates to HFOs [24, 36], demonstrating its versatility and reliability as a preparatory tool for comprehensive compositional analysis of complex hydrocarbon mixtures.

## Author Contributions

**Kawthar Z. Alzarieni** was responsible for conceptualizing the study, performing the experiments, and writing the manuscript. **Wan Tang** **Jeff Zhang** contributed to the software processing of data. **Brent Modereger** and **Wanru Li** assisted in method development. **Gozdem Kilaz** provided valuable insights and access to instrumentation. **Hilkka I. Kenttämaa** supervised the project, secured funding, and contributed to writing, reviewing, and editing the manuscript.

## Funding

The authors express gratitude for the generous funding provided by NESTE and appreciate their contribution in supplying samples and insights during the analysis. Kawthar Alzarieni extends her sincere thanks to the Jordanian Ministry of Higher Education and Scientific Research/Scientific Research and Innovation Support Fund for awarding her a postdoctoral fellowship and supporting her research stay at Purdue University.

## Conflicts of Interest

The authors declare no conflicts of interest.

## Supporting information




**Supporting File 1**: jssc70341‐sup‐0001‐FigureS1.png

**Supporting File 2**: jssc70341‐sup‐0002‐FigureS2.png

**Supporting File 3**: jssc70341‐sup‐0003‐TableS1.docx

**Supporting File 4**: jssc70341‐sup‐0004‐TableS2.docx

**Supporting File 5**: jssc70341‐sup‐0005‐TableS3.docx

**Supporting File 6**: jssc70341‐sup‐0006‐SuppMat.docx

## Data Availability

The authors confirm that the data supporting the findings of this study are available within the article and/or its supporting information.
