# Supplementary figures and images for: Determination of the Weight Percent of Aromatic Compounds in a Heavy Fuel Oil by Using Flash Chromatography and Solid‐phase Extraction Coupled With High‐Temperature Two‐Dimensional Gas Chromatography and Electron Ionization Time‐of‐Flight High‐Resolution Mass Spectrometry
Source: J Sep Sci. 2025 Dec 28;48(12):e70341. doi: 10.1002/jssc.70341 (PMC12745910; doi:10.1002/jssc.70341)

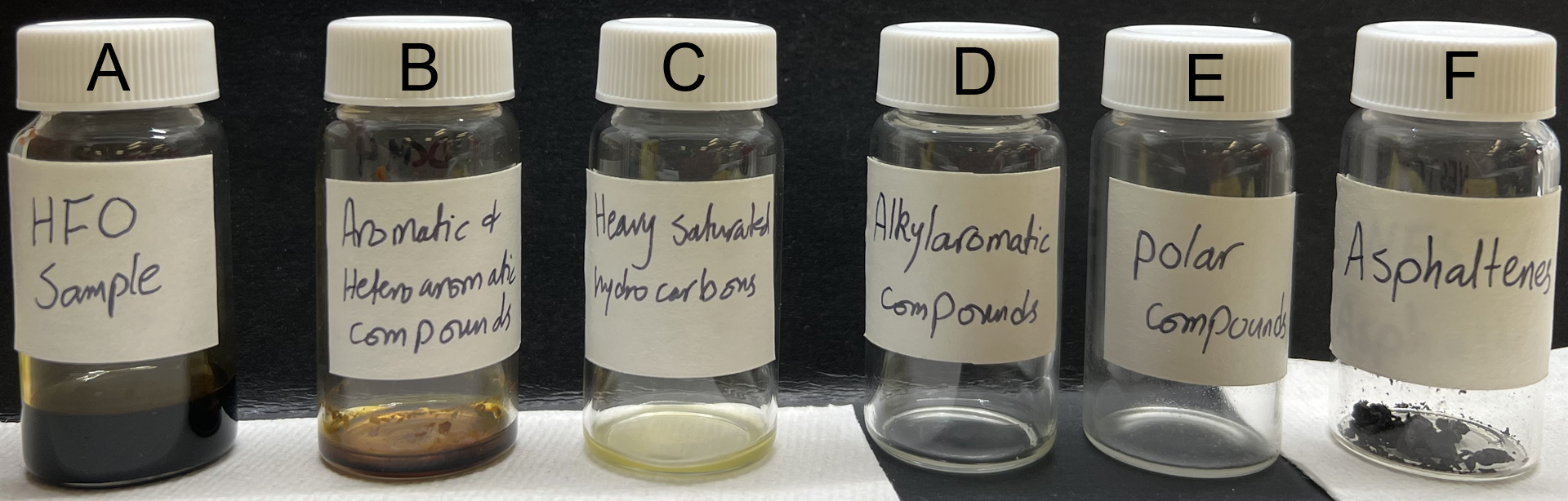

Supplement: Supplementary file 1 — Supporting File 1: jssc70341‐sup‐0001‐FigureS1.png [file JSSC-48-e70341-s004.png]

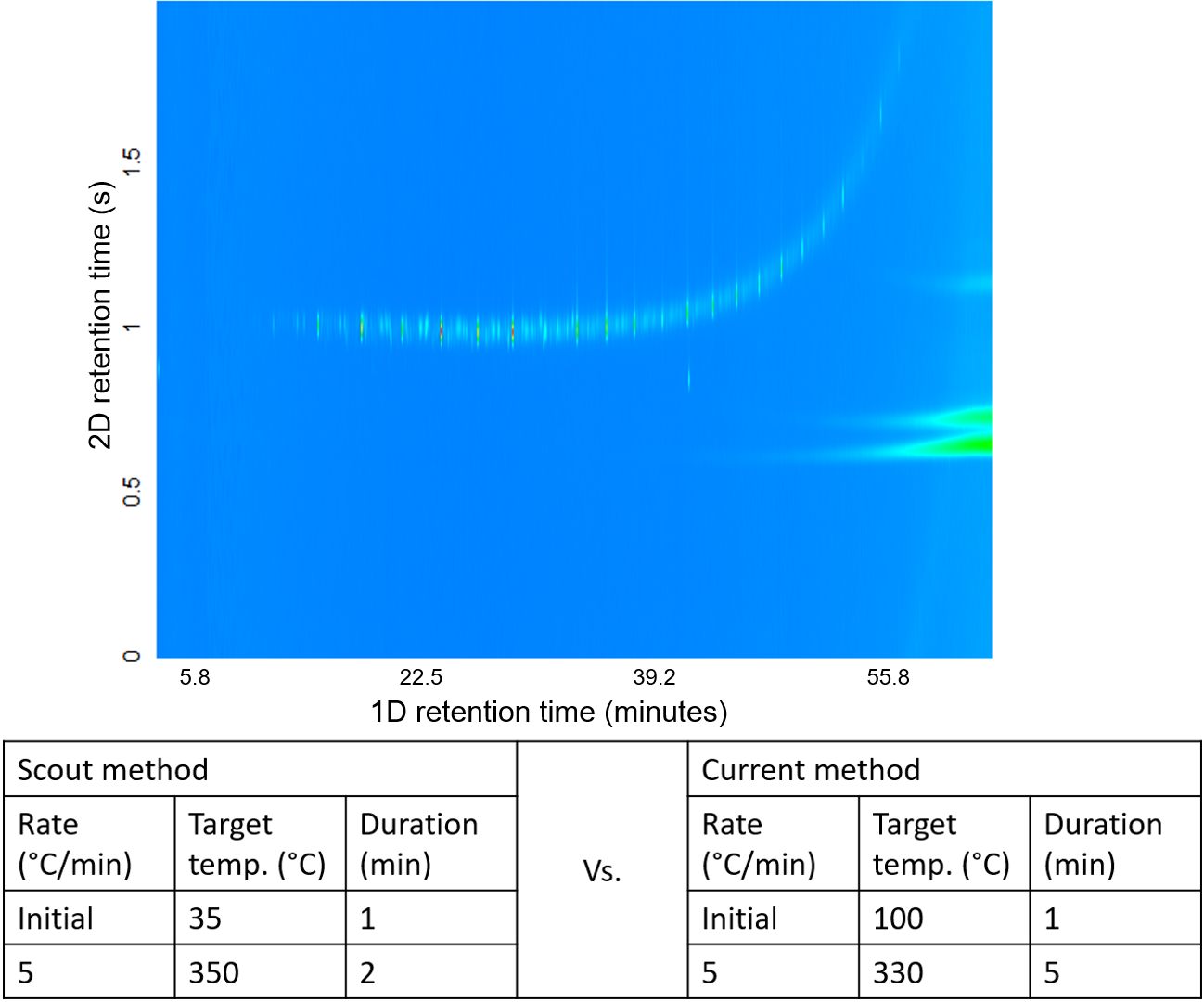

Supplement: Supplementary file 2 — Supporting File 2: jssc70341‐sup‐0002‐FigureS2.png [file JSSC-48-e70341-s005.png]
