# Supplementary material for: Determination of the Weight Percent of Aromatic Compounds in a Heavy Fuel Oil by Using Flash Chromatography and Solid‐phase Extraction Coupled With High‐Temperature Two‐Dimensional Gas Chromatography and Electron Ionization Time‐of‐Flight High‐Resolution Mass Spectrometry
Source: J Sep Sci. 2025 Dec 28;48(12):e70341. doi: 10.1002/jssc.70341 (PMC12745910; doi:10.1002/jssc.70341)
Supplement: Supplementary file 3 — Supporting File 3: jssc70341‐sup‐0003‐TableS1.docx [file JSSC-48-e70341-s002.docx]

**Table S1**

| Fractionation method | |
| --- | --- |
| Sample mass | 300 mg |
| Column packing mass | 40 g |
| Solvent flow rate | 40 mLmin^-1^ |
| Volume used of each solvent | 400 mL |
| Average % recovery | 98.4±0.8% |
| Solid phase extraction (SPE) | |
| Sample mass | 80 mg |
| Column packing mass | 1.5 g |
| Solvent flow rate | 1 mLmin^-1^ |
| Volume used of each solvent | 5 mL |
| Average % recovery | 98.0±0.1% |
