# Supplementary material for: Determination of the Weight Percent of Aromatic Compounds in a Heavy Fuel Oil by Using Flash Chromatography and Solid‐phase Extraction Coupled With High‐Temperature Two‐Dimensional Gas Chromatography and Electron Ionization Time‐of‐Flight High‐Resolution Mass Spectrometry
Source: J Sep Sci. 2025 Dec 28;48(12):e70341. doi: 10.1002/jssc.70341 (PMC12745910; doi:10.1002/jssc.70341)
Supplement: Supplementary file 4 — Supporting File 4: jssc70341‐sup‐0004‐TableS2.docx [file JSSC-48-e70341-s001.docx]

**Table S2**

| Peak # | Compound name | Formula | Expected Ion *m/z* | Observed Ion *m/z* | Peak area | S/N | Retention times in first and second dimensions (s) |
| --- | --- | --- | --- | --- | --- | --- | --- |
| Solvent peak | Hexane | C_6_H_14_ | 86.1 | 86.1 |  | | 447.352, 1.723 |
| 1 | 2,6,6-trimethylbicyclo[3.1.1]heptane | C_10_H_18_ | 138.1 | 138.1 | 39326 | 118 | 2724.46, 2.474 |
| 2 | Dodecane | C_12_H_26_ | 170.2 | 156.2 | 343704 | 220 | 2689.47, 2.540 |
| 3 | Tridecane | C_13_H_28_ | 184.2 | 114.2 | 53608 | 195 | 664.928, 2.299 |
| 4 | Tetradecane | C_14_H_30_ | 198.2 | 126.1 | 125603 | 140 | 869.882, 2.397 |
| 5 | 1-ethyl-3-heptylcyclohexane | C_15_H_30_ | 210.2 | 124.1 | 136927 | 404 | 809.895, 2.340 |
| 6 | Pentadecane | C_15_H_32_ | 212.2 | 113.1 | 155599 | 462 | 954.863, 2.361 |
| 7 | 2,6,10-trimethyltridecane | C_16_H_34_ | 226.3 | 85.1 | 25590 | 95 | 534.958, 2.190 |
| 8 | 5-ethyltetradecane | C_16_H_34_ | 226.3 | 99.1 | 110940 | 326 | 1014.85, 2.396 |
| 9 | Hexadecane | C_16_H_34_ | 226.3 | 141.2 | 287639 | 736 | 1094.83, 2.371 |
| 10 | Heptadecane | C_17_H_36_ | 240.3 | 155.2 | 274647 | 422 | 1234.8, 2.361 |
| 11 | Octadecane | C_18_H_38_ | 254.3 | 112.1 | 146226 | 458 | 1284.79, 2.391 |
| 12 | 7,9-dimethylhexadecane | C_18_H_38_ | 254.3 | 141.2 | 277853 | 606 | 1364.77, 2.361 |
| 13 | Nonadecane | C_19_H_40_ | 268.3 | 169.2 | 235772 | 576 | 1494.74, 2.345 |
| 14 | Icosane | C_20_H_42_ | 282.3 | 155.2 | 229228 | 585 | 1614.71, 2.345 |
| 15 | Henicosane | C_21_H_44_ | 296.3 | 141.2 | 280847 | 475 | 1734.69, 2.330 |
| 16 | Docosane | C_22_H_46_ | 310.4 | 183.2 | 343865 | 700 | 1844.66, 2.335 |
| 17 | Tricosane | C_23_H_48_ | 324.4 | 183.2 | 360265 | 625 | 1949.64, 2.345 |
| 18 | Tetracosane | C_24_H_50_ | 338.4 | 169.2 | 399824 | 575 | 2054.61, 2.345 |
| 19 | Pentacosane | C_25_H_52_ | 352.4 | 155.2 | 405566 | 520 | 2149.59, 2.366 |
| 20 | 4,6,17-trimethyltricosane | C_26_H_54_ | 366.4 | 183.2 | 340906 | 407 | 2244.57, 2.376 |
| 21 | 4,6,12,17-tetramethyltricosane | C_27_H_56_ | 380.4 | 155.2 | 343374 | 352 | 2339.55, 2.386 |
| 22 | 4,6,12,17-tetramethyltetracosane | C_28_H_58_ | 394.5 | 155.2 | 359045 | 345 | 2424.53, 2.417 |
| 23 | 11-heptyldocosane | C_29_H_60_ | 408.5 | 169.2 | 467485 | 288 | 2509.51, 2.438 |
| 24 | Triacontane | C_30_H_62_ | 422.5 | 169.2 | 551404 | 326 | 2594.49, 2.463 |
| 25 | 3,5,24-trimethyloctacosane | C_31_H_64_ | 436.5 | 197.2 | 724848 | 363 | 2674.47, 2.496 |
| 26 | Dotriacontane | C_32_H_66_ | 450.5 | 197.2 | 634930 | 345 | 2749.45, 2.541 |
| 27 | Hentriacontane | C_31_H_64_ | 436.5 | 211.2 | 509022 | 279 | 2824.44, 2.623 |
| 28 | Tetratriacontane | C_34_H_70_ | 478.5 | 197.2 | 687627 | 312 | 2904.42, 2.891 |
| 29 | Pentatriacontane | C_35_H_72_ | 492.6 | 183.2 | 819168 | 288 | 2989.4, 3.183 |
| 30 | Hexatriacontane | C_36_H_74_ | 506.6 | 197.2 | 859048 | 248 | 3084.38, 3.523 |
