# Supplementary material for: Determination of the Weight Percent of Aromatic Compounds in a Heavy Fuel Oil by Using Flash Chromatography and Solid‐phase Extraction Coupled With High‐Temperature Two‐Dimensional Gas Chromatography and Electron Ionization Time‐of‐Flight High‐Resolution Mass Spectrometry
Source: J Sep Sci. 2025 Dec 28;48(12):e70341. doi: 10.1002/jssc.70341 (PMC12745910; doi:10.1002/jssc.70341)
Supplement: Supplementary file 5 — Supporting File 5: jssc70341‐sup‐0005‐TableS3.docx [file JSSC-48-e70341-s003.docx]

**Table S3**

| Peak # | Compound name | Formula | Expected Ion *m/z* | Observed Ion *m/z* | Peak area | S/N | RDBE | Retention times in first and second dimensions (s) |
| --- | --- | --- | --- | --- | --- | --- | --- | --- |
| Solvent peak | Isopropyl alcohol | C_3_H_8_O | 60.1 | 60.1 |  | |  | 619.6,1.235 |
| 1 | 1-Thiaphenalene | C_12_H_8_S | 184.2 | 184.0 | 35103 | 152 | 9 | 1664.7, 1.795 |
| 2 | 1H-Phenalen-1-one | C_13_H_8_O | 180.2 | 180.1 | 39022 | 169 | 10 | 1934.64, 1.723 |
| 3 | 2-Fluorenecarboxaldehyde | C_14_H_10_O | 194.2 | 194.1 | 42195 | 143 | 10 | 1979.63, 1.754 |
| 4 | 5-pentyl-4,5,6,7-tetrahydro-1H-indene-2-carboxamide | C_15_H_23_NO | 233.2 | 233.2 | 23989 | 104 | 5 | 2024.62, 1.882 |
| 5 | O6-(2-hydroxyethyl)guanine | C_7_H_9_N_5_O_2_ | 195.2 | 195.1 | 26021 | 112 | 8.5 | 2164.59, 1.764 |
| 6 | 7-(pentan-2-yl)-5-tridecyl-1H-indene-2-carboxamide | C_28_H_45_NO | 411.3 | 282.1 | 76417 | 327 | 7 | 2234.57, 1.918 |
| 7 | Hexa(methoxymethyl)melamine | C_15_H_30_N_6_O_6_ | 390.4 | 359.2 | 137081 | 356 | 7.5 | 2354.54, 1.887 |
| 8 | 5-propyl-1,2l4-oxathiole | C_6_H_10_OS | 130.0 | 130.0 | 161158 | 692 | 2 |  |
| 9 | 5-isobutyl-1,2l4-oxathiole | C_7_H_12_OS | 144.1 | 144.1 | 25497 | 111 | 2 | 2249.57, 2.092 |
| 10 | 2,3-dihydro-Benzofuran | C_8_H_8_O | 120.1 | 120.1 | 36577 | 159 | 5 | 2584.49, 1.857 |
