# Supplementary material for: Determination of the Weight Percent of Aromatic Compounds in a Heavy Fuel Oil by Using Flash Chromatography and Solid‐phase Extraction Coupled With High‐Temperature Two‐Dimensional Gas Chromatography and Electron Ionization Time‐of‐Flight High‐Resolution Mass Spectrometry
Source: J Sep Sci. 2025 Dec 28;48(12):e70341. doi: 10.1002/jssc.70341 (PMC12745910; doi:10.1002/jssc.70341)
Supplement: Supplementary file 6 — Supporting File 6: jssc70341‐sup‐0006‐SuppMat.docx [file JSSC-48-e70341-s006.docx]

**Supporting Information**

**Determination of the Weight Percent of Aromatic Compounds in a Heavy Fuel Oil by Using Flash Chromatography and Solid-phase Extraction Coupled with High-temperature Two-dimensional Gas Chromatography and Electron Ionization Time-of-flight High-resolution Mass Spectrometry**

Kawthar Z. Alzarieni,^a^ Wan Tang Jeff Zhang,^b^ Brent Modereger,^b^ Wanru Li,^b^ Gozdem Kilaz,^c^ Hilkka I. Kenttämaa^b,*^

^a^ Jordan University of Science & Technology, Faculty of Pharmacy, Department of Medicinal Chemistry and Pharmacognosy, Irbid 22110, Jordan

^b^ James Tarpo Jr. and Margaret Tarpo Department of Chemistry, Purdue University, West Lafayette, IN, USA, 47907

^c^ Department of Engineering Technology, Purdue University, West Lafayette, IN 47907

^*^Corresponding author:

Hilkka I. Kenttämaa

Address: Department of Chemistry, Purdue University, West Lafayette, IN 47907, USA.

Tel.: +1 (765) 494 0882; fax: +1 (765) 494 9421.

E-mail: [hilkka@purdue.edu](mailto:hilkka@purdue.edu)

**List of contents**

**Table S1*.*** Key parameters for the fractionation methods, including sample and column masses, solvent flow rates, volumes, and average recoveries, illustrating method efficiency and reproducibility…………………………………………………………………………………..**S3**

**Figure S1*.*** Visual comparison of the HFO sample and its separated fractions, highlighting their distinct colors: A) bulk HFO (dark black), B) aromatic and heteroaromatic fraction (reddish brown), C) heavy saturated hydrocarbons (light yellow), D) alkylaromatics (colorless/transparent), E) polar compounds (white residue), and F) asphaltenes (black solid). ………………………………………………………………………………………………………..**S3**

**Figure S2.** GC × GC chromatogram of the heavy saturated hydrocarbon fraction acquired during method development using a scout method (300 °C inlet, 35 °C starting oven temperature, 350 °C final oven temperature). The largest analyte eluted at 3550.92 s (55.85 min) in the first dimension and 1.82 s in the second dimension (~325 °C oven temperature), supporting the choice of 330 °C as the final oven temperature in the optimized method. Differences between the GC × GC scout method and the final optimized method are indicated……………………………………………………………………………………………..**S4**

**Table S2.** Data obtained for compounds in the saturated hydrocarbon fraction by using GC$\times$GC/EI TOF HRMS. Data provided are based on ChromaTOF software prediction of the molecular ion *m/z* value and the identification of the compound by using the EI mass spectral library match using a match factor threshold of 800. Retention times of model compounds aided in identifying higher molecular weight species and improving confidence in compound assignments.……..……………………………………………………….…………………………**S5**

**Table S3.** Data obtained for compounds in the polar fraction by using GC$\times$GC/EI TOF HRMS. Data provided are based on ChromaTOF software prediction of the molecular ion *m/z* value and the identification of the compound by using the EI mass spectral library match using a match factor threshold of 800………………………………………………………….…………...**S6**

**Table S1**. Key parameters for the fractionation methods, including sample and column masses, solvent flow rates, volumes, and average recoveries, illustrating method efficiency and reproducibility.

| Fractionation method | |
| --- | --- |
| Sample mass | 300 mg |
| Column packing mass | 40 g |
| Solvent flow rate | 40 mLmin^-1^ |
| Volume used of each solvent | 400 mL |
| Average % recovery | 98.4±0.8% |
| Solid phase extraction (SPE) | |
| Sample mass | 80 mg |
| Column packing mass | 1.5 g |
| Solvent flow rate | 1 mLmin^-1^ |
| Volume used of each solvent | 5 mL |
| Average % recovery | 98.0±0.1% |


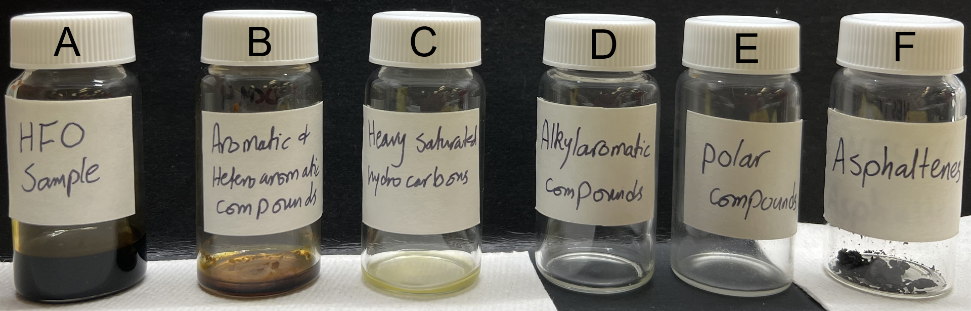


**Figure S1**. Visual comparison of the HFO sample and its separated fractions, highlighting their distinct colors: A) bulk HFO (dark black), B) aromatic and heteroaromatic fraction (reddish brown), C) heavy saturated hydrocarbons (light yellow), D) alkylaromatics (colorless/transparent), E) polar compounds (white residue), and F) asphaltenes (black solid).


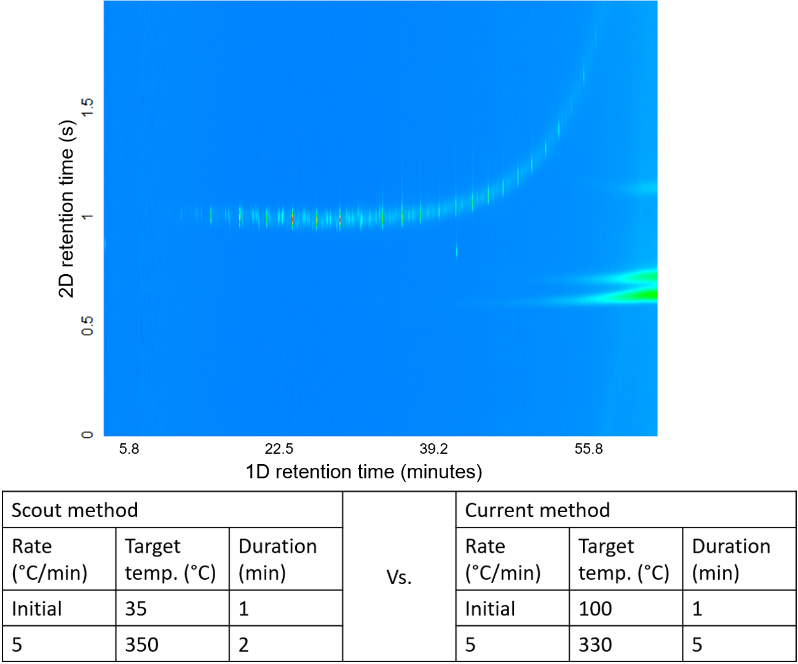


**Figure S2**. GC × GC chromatogram of the heavy saturated hydrocarbon fraction acquired during method development using a scout method (300 °C inlet, 35 °C starting oven temperature, 350 °C final oven temperature). The largest analyte eluted at 3550.92 s (55.85 min) in the first dimension and 1.82 s in the second dimension (~325 °C oven temperature), supporting the choice of 330 °C as the final oven temperature in the optimized method. Differences between the GC × GC scout method and the final optimized method are indicated.

**Table S2**. Data obtained for compounds in the saturated hydrocarbon fraction by using GC × GC/EI TOF HRMS. Data provided are based on ChromaTOF software prediction of the molecular ion m/z value and the identification of the compound by using the EI mass spectral library match using a match factor threshold of 800. Retention times of model compounds aided in identifying higher molecular weight species and improving confidence in compound assignments.

| Peak # | Compound name | Formula | Expected Ion *m/z* | Observed Ion *m/z* | Peak area | S/N | Retention times in first and second dimensions (s) |
| --- | --- | --- | --- | --- | --- | --- | --- |
| Solvent peak | Hexane | C_6_H_14_ | 86.1 | 86.1 |  | | 447.352, 1.723 |
| 1 | 2,6,6-trimethylbicyclo[3.1.1]heptane | C_10_H_18_ | 138.1 | 138.1 | 39326 | 118 | 2724.46, 2.474 |
| 2 | Dodecane | C_12_H_26_ | 170.2 | 156.2 | 343704 | 220 | 2689.47, 2.540 |
| 3 | Tridecane | C_13_H_28_ | 184.2 | 114.2 | 53608 | 195 | 664.928, 2.299 |
| 4 | Tetradecane | C_14_H_30_ | 198.2 | 126.1 | 125603 | 140 | 869.882, 2.397 |
| 5 | 1-ethyl-3-heptylcyclohexane | C_15_H_30_ | 210.2 | 124.1 | 136927 | 404 | 809.895, 2.340 |
| 6 | Pentadecane | C_15_H_32_ | 212.2 | 113.1 | 155599 | 462 | 954.863, 2.361 |
| 7 | 2,6,10-trimethyltridecane | C_16_H_34_ | 226.3 | 85.1 | 25590 | 95 | 534.958, 2.190 |
| 8 | 5-ethyltetradecane | C_16_H_34_ | 226.3 | 99.1 | 110940 | 326 | 1014.85, 2.396 |
| 9 | Hexadecane | C_16_H_34_ | 226.3 | 141.2 | 287639 | 736 | 1094.83, 2.371 |
| 10 | Heptadecane | C_17_H_36_ | 240.3 | 155.2 | 274647 | 422 | 1234.8, 2.361 |
| 11 | Octadecane | C_18_H_38_ | 254.3 | 112.1 | 146226 | 458 | 1284.79, 2.391 |
| 12 | 7,9-dimethylhexadecane | C_18_H_38_ | 254.3 | 141.2 | 277853 | 606 | 1364.77, 2.361 |
| 13 | Nonadecane | C_19_H_40_ | 268.3 | 169.2 | 235772 | 576 | 1494.74, 2.345 |
| 14 | Icosane | C_20_H_42_ | 282.3 | 155.2 | 229228 | 585 | 1614.71, 2.345 |
| 15 | Henicosane | C_21_H_44_ | 296.3 | 141.2 | 280847 | 475 | 1734.69, 2.330 |
| 16 | Docosane | C_22_H_46_ | 310.4 | 183.2 | 343865 | 700 | 1844.66, 2.335 |
| 17 | Tricosane | C_23_H_48_ | 324.4 | 183.2 | 360265 | 625 | 1949.64, 2.345 |
| 18 | Tetracosane | C_24_H_50_ | 338.4 | 169.2 | 399824 | 575 | 2054.61, 2.345 |
| 19 | Pentacosane | C_25_H_52_ | 352.4 | 155.2 | 405566 | 520 | 2149.59, 2.366 |
| 20 | 4,6,17-trimethyltricosane | C_26_H_54_ | 366.4 | 183.2 | 340906 | 407 | 2244.57, 2.376 |
| 21 | 4,6,12,17-tetramethyltricosane | C_27_H_56_ | 380.4 | 155.2 | 343374 | 352 | 2339.55, 2.386 |
| 22 | 4,6,12,17-tetramethyltetracosane | C_28_H_58_ | 394.5 | 155.2 | 359045 | 345 | 2424.53, 2.417 |
| 23 | 11-heptyldocosane | C_29_H_60_ | 408.5 | 169.2 | 467485 | 288 | 2509.51, 2.438 |
| 24 | Triacontane | C_30_H_62_ | 422.5 | 169.2 | 551404 | 326 | 2594.49, 2.463 |
| 25 | 3,5,24-trimethyloctacosane | C_31_H_64_ | 436.5 | 197.2 | 724848 | 363 | 2674.47, 2.496 |
| 26 | Dotriacontane | C_32_H_66_ | 450.5 | 197.2 | 634930 | 345 | 2749.45, 2.541 |
| 27 | Hentriacontane | C_31_H_64_ | 436.5 | 211.2 | 509022 | 279 | 2824.44, 2.623 |
| 28 | Tetratriacontane | C_34_H_70_ | 478.5 | 197.2 | 687627 | 312 | 2904.42, 2.891 |
| 29 | Pentatriacontane | C_35_H_72_ | 492.6 | 183.2 | 819168 | 288 | 2989.4, 3.183 |
| 30 | Hexatriacontane | C_36_H_74_ | 506.6 | 197.2 | 859048 | 248 | 3084.38, 3.523 |

**Table S3**. Data obtained for compounds in the polar fraction by using GC × GC/EI TOF HRMS. Data provided are based on ChromaTOF software prediction of the molecular ion m/z value and the identification of the compound by using the EI mass spectral library match using a match factor threshold of 800.

| Peak # | Compound name | Formula | Expected Ion *m/z* | Observed Ion *m/z* | Peak area | S/N | RDBE | Retention times in first and second dimensions (s) |
| --- | --- | --- | --- | --- | --- | --- | --- | --- |
| Solvent peak | Isopropyl alcohol | C_3_H_8_O | 60.1 | 60.1 |  | |  | 619.6,1.235 |
| 1 | 1-Thiaphenalene | C_12_H_8_S | 184.2 | 184.0 | 35103 | 152 | 9 | 1664.7, 1.795 |
| 2 | 1H-Phenalen-1-one | C_13_H_8_O | 180.2 | 180.1 | 39022 | 169 | 10 | 1934.64, 1.723 |
| 3 | 2-Fluorenecarboxaldehyde | C_14_H_10_O | 194.2 | 194.1 | 42195 | 143 | 10 | 1979.63, 1.754 |
| 4 | 5-pentyl-4,5,6,7-tetrahydro-1H-indene-2-carboxamide | C_15_H_23_NO | 233.2 | 233.2 | 23989 | 104 | 5 | 2024.62, 1.882 |
| 5 | O6-(2-hydroxyethyl)guanine | C_7_H_9_N_5_O_2_ | 195.2 | 195.1 | 26021 | 112 | 8.5 | 2164.59, 1.764 |
| 6 | 7-(pentan-2-yl)-5-tridecyl-1H-indene-2-carboxamide | C_28_H_45_NO | 411.3 | 282.1 | 76417 | 327 | 7 | 2234.57, 1.918 |
| 7 | Hexa(methoxymethyl)melamine | C_15_H_30_N_6_O_6_ | 390.4 | 359.2 | 137081 | 356 | 7.5 | 2354.54, 1.887 |
| 8 | 5-propyl-1,2l4-oxathiole | C_6_H_10_OS | 130.0 | 130.0 | 161158 | 692 | 2 |  |
| 9 | 5-isobutyl-1,2l4-oxathiole | C_7_H_12_OS | 144.1 | 144.1 | 25497 | 111 | 2 | 2249.57, 2.092 |
| 10 | 2,3-dihydro-Benzofuran | C_8_H_8_O | 120.1 | 120.1 | 36577 | 159 | 5 | 2584.49, 1.857 |
